# 2-Amino-4-(4-chloro­phen­yl)-5-oxo-5,6,7,8-tetra­hydro-4*H*-chromene-3-carbonitrile

**DOI:** 10.1107/S1600536812015838

**Published:** 2012-04-18

**Authors:** Shaaban K. Mohamed, Mehmet Akkurt, Antar A. Abdelhamid, Kuldip Singh, M. A. Allahverdiyev

**Affiliations:** a Chemistry and Environmental Division, Manchester Metropolitan University, Manchester M1 5GD, England; b Department of Physics, Faculty of Sciences, Erciyes University, 38039 Kayseri, Turkey; c Department of Chemistry, University of Leicester, Leicester, England; d Department of Organic Chemistry, Baku State University, Baku, Azerbaijan

## Abstract

In the title moleclue, C_16_H_13_ClN_2_O_2_, the cyclo­hexene ring is in a sofa conformation. The pyran ring is essentialy planar [maximum deviation = 0.038 (2) Å] and forms a dihedral angle of 89.68 (10)° with the benzene ring. In the crystal, mol­ecules are linked by pairs of N—H⋯N hydrogen bonds, forming inversion dimers with *R*_2_^2^(12) ring motifs. These dimers are further linked by N—H⋯O hydrogen bonds into chains along [110]. Weak C—H⋯O hydrogen bonds are also present.

## Related literature

For pharmaceutical background to 2-amino-5-oxo-5,6,7,8-tetra­hydro-4*H*-chromene-3-carbonitrile derivatives, see: Gao *et al.* (2001 ▶); Xu *et al.* (2011 ▶); Luan *et al.* (2011 ▶); Wang & Zhu, (2007 ▶); O’Callaghan *et al.* (1995 ▶). For similar structures, see: Tu *et al.* (2001 ▶); Qiao *et al.* (2011 ▶); Kong *et al.* (2011 ▶); Hu *et al.* (2012 ▶). For standard bond lengths, see: Allen *et al.* (1987 ▶). For geometric analysis, see: Cremer & Pople (1975 ▶). For hydrogen-bond motifs, see: Bernstein *et al.* (1995 ▶); Etter *et al.* (1990 ▶).
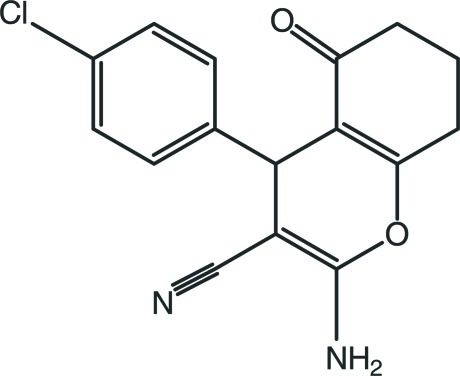


## Experimental

### 

#### Crystal data

C_16_H_13_ClN_2_O_2_
*M* *_r_* = 300.73
Monoclinic, 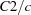

*a* = 13.753 (4) Å
*b* = 11.077 (3) Å
*c* = 19.370 (6) Å
β = 107.856 (5)°
*V* = 2808.7 (14) Å^3^
*Z* = 8
Mo *K*α radiation
μ = 0.28 mm^−1^
*T* = 150 K
0.43 × 0.27 × 0.07 mm

#### Data collection

Bruker APEX 2000 CCD area-detector diffractometer
Absorption correction: multi-scan (*SADABS*; Sheldrick, 1996 ▶) *T*_min_ = 0.914, *T*_max_ = 0.981
10643 measured reflections
2755 independent reflections
2124 reflections with *I* > 2σ(*I*)
*R*_int_ = 0.063

#### Refinement

*R*[*F* ^2^ > 2σ(*F* ^2^)] = 0.049
*wR*(*F* ^2^) = 0.122
*S* = 1.02
2755 reflections
190 parameters
H-atom parameters constrained
Δρ_max_ = 0.35 e Å^−3^
Δρ_min_ = −0.23 e Å^−3^

### 

Data collection: *APEX2* (Bruker, 2005 ▶); cell refinement: *SAINT* (Bruker, 2005 ▶); data reduction: *SAINT*; program(s) used to solve structure: *SHELXS97* (Sheldrick, 2008 ▶); program(s) used to refine structure: *SHELXL97* (Sheldrick, 2008 ▶); molecular graphics: *ORTEP-3 for Windows* (Farrugia, 1997 ▶) and *PLATON* (Spek, 2009 ▶); software used to prepare material for publication: *WinGX* (Farrugia, 1999 ▶) and *PLATON*.

## Supplementary Material

Crystal structure: contains datablock(s) global, I. DOI: 10.1107/S1600536812015838/lh5452sup1.cif

Structure factors: contains datablock(s) I. DOI: 10.1107/S1600536812015838/lh5452Isup2.hkl

Supplementary material file. DOI: 10.1107/S1600536812015838/lh5452Isup3.cml

Additional supplementary materials:  crystallographic information; 3D view; checkCIF report

## Figures and Tables

**Table 1:** Hydrogen-bond geometry (Å, °)

*D*—H⋯*A*	*D*—H	H⋯*A*	*D*⋯*A*	*D*—H⋯*A*
N1—H1*A*⋯N2^i^	0.88	2.25	3.132 (3)	177
N1—H1*B*⋯O1^ii^	0.88	2.15	2.955 (2)	151
C3—H3⋯N2^iii^	0.95	2.48	3.226 (3)	135

Symmetry codes: (i) 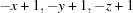; (ii) 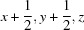; (iii) 
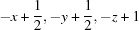
.

**Fig. 1.**
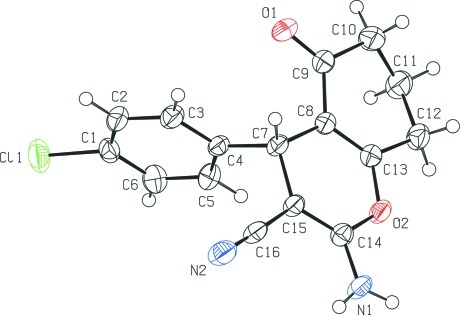
A view of the molecule of (I), with displacement ellipsoids for non-H atoms drawn at the 50% probability level.

**Fig. 2.**
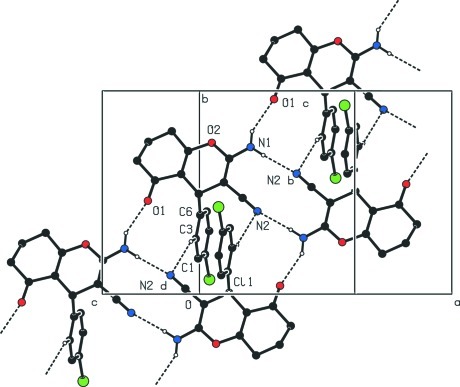
Part of the crystal structure of (I) with hydrogen bonds shown as dashed lines. H atoms not involved in H-bonding are omitted for clarity. Symmetry codes: (b) -x+1, -y+1, -z+1; (c) x+1/2, y+1/2, z; (d) -x+1/2, -y+1/2, -z+1.

## supplementary crystallographic information

### Comment

The existence of an amino group and cyano group in tetrahydrochromenone compounds make them great substrates for building up multi organic transformations (Luan *et al.*, 2011), preparing poly-functionalized substituted pyran derivatives (Wang & Zhu, 2007) and designing of poly-heterocyclic compounds (O'Callaghan *et al.*,1995). Moreover, such derivatives of tetrahydrochromenones have attracted strong interests of pharmacists and biologists because of their potential application in the treatment of psoriatic arthritis and rheumatoid arthritis (Xu *et al.*, 2011). They also have wide biological applications such as anti-anaphylaxis, anti-achondroplasty and anti-cancer activity (Gao *et al*., 2001). To continue to our interest in the synthesis of biologically active compounds we report herein the crystal structure of the title compound.

The molecular structure of the title compound is shown in Fig. 1. The (C8–C13) cyclohexene ring is in a sofa conformation with puckering parameters (Cremer & Pople, 1975) of Q_T_ = 0.466 (3) Å, θ = 58.3 (2) ° and φ = 173.5 (3) °. The pyran ring (O2/C7/C8/C13—C15) is essentialy planar with a maximum deviation of 0.038 (2) Å for C7, and forms a dihedral angle of 89.68 (10)° with the benzene ring (C1-C6). The bond lengths (Allen *et al.*, 1987) and angles are similar to those for reported structures (Tu *et al.*, 2001; Qiao *et al.*, 2011; Kong *et al.*, 2011; Hu *et al.*, 2012).

In the crystal, molecules are linked by pairs of intermolecular N—H···N hydrogen bonds, forming inversion dimers with *R*_2_^2^(12) ring motifs (Bernstein *et al.*, 1995; Etter *et al.*, 1990), and these dimers are connected by weak C—H···N and N—H···O hydrogen bonds, generating one-dimensional chains along [110] (Table 1, Fig. 2).

### Experimental

The title compound (I) was formed during a three component reaction of an equimolar ratios of (4-chlorobenzylidene)propanedinitrile (1 mmol), (4-aminophenyl)methanol (1 mmol) and cyclohexane-1,3-dione (1 mmol). The reaction mixture was heated in ethanol at 351 K. The reaction was monitored with TLC until completed after 5 h, then left in fume cupboard at room temperature until solvent evaporated. The resulting solid mass was recrystallized from ethanol to afford good quality crystals suitable for X-ray diffraction. [Yield: 83%, m.p.: 513 K].

### Refinement

All H atoms were positioned geometrically and refined using as riding model with N—H = 0.88 Å for NH_2_, C—H = 0.95 Å for aromatic, C—H = 0.99 Å for methylene and C—H = 1.00 Å for methine, and with *U*_iso_(H) = 1.2*U*_eq_(C,*N*)

### Crystal data

**Table:**

C_16_H_13_ClN_2_O_2_	*F*(000) = 1248
*M**_r_* = 300.73	*D*_x_ = 1.422 Mg m^−^^3^
Monoclinic, *C*2/*c*	Mo *K*α radiation, λ = 0.71073 Å
Hall symbol: -C 2yc	Cell parameters from 833 reflections
*a* = 13.753 (4) Å	θ = 2.2–28.2°
*b* = 11.077 (3) Å	µ = 0.28 mm^−^^1^
*c* = 19.370 (6) Å	*T* = 150 K
β = 107.856 (5)°	Plate, colourless
*V* = 2808.7 (14) Å^3^	0.43 × 0.27 × 0.07 mm
*Z* = 8	

### Data collection

**Table:**

Bruker APEX 2000 CCD area-detector diffractometer	2755 independent reflections
Radiation source: fine-focus sealed tube	2124 reflections with *I* > 2σ(*I*)
Graphite monochromator	*R*_int_ = 0.063
phi and ω scans	θ_max_ = 26.0°, θ_min_ = 2.2°
Absorption correction: multi-scan (*SADABS*; Sheldrick, 1996)	*h* = −16→16
*T*_min_ = 0.914, *T*_max_ = 0.981	*k* = −13→13
10643 measured reflections	*l* = −23→23

### Refinement

**Table:**

Refinement on *F*^2^	Primary atom site location: structure-invariant direct methods
Least-squares matrix: full	Secondary atom site location: difference Fourier map
*R*[*F*^2^ > 2σ(*F*^2^)] = 0.049	Hydrogen site location: inferred from neighbouring sites
*wR*(*F*^2^) = 0.122	H-atom parameters constrained
*S* = 1.02	*w* = 1/[σ^2^(*F*_o_^2^) + (0.0649*P*)^2^] where *P* = (*F*_o_^2^ + 2*F*_c_^2^)/3
2755 reflections	(Δ/σ)_max_ < 0.001
190 parameters	Δρ_max_ = 0.35 e Å^−^^3^
0 restraints	Δρ_min_ = −0.23 e Å^−^^3^

### Special details

**Table:**

Geometry. Bond distances, angles *etc*. have been calculated using the rounded fractional coordinates. All su's are estimated from the variances of the (full) variance-covariance matrix. The cell e.s.d.'s are taken into account in the estimation of distances, angles and torsion angles
Refinement. Refinement on *F*^2^ for ALL reflections except those flagged by the user for potential systematic errors. Weighted *R*-factors *wR* and all goodnesses of fit *S* are based on *F*^2^, conventional *R*-factors *R* are based on *F*, with *F* set to zero for negative *F*^2^. The observed criterion of *F*^2^ > σ(*F*^2^) is used only for calculating -*R*-factor-obs *etc*. and is not relevant to the choice of reflections for refinement. *R*-factors based on *F*^2^ are statistically about twice as large as those based on *F*, and *R*-factors based on ALL data will be even larger.

### Fractional atomic coordinates and isotropic or equivalent isotropic displacement parameters (Å^2^)

**Table:**

	*x*	*y*	*z*	*U*_iso_*/*U*_eq_	
Cl1	0.12556 (5)	0.07141 (5)	0.22343 (4)	0.0510 (2)	
O1	−0.04515 (11)	0.45923 (13)	0.41373 (8)	0.0395 (5)	
O2	0.20240 (10)	0.74455 (12)	0.39806 (8)	0.0320 (4)	
N1	0.37218 (13)	0.72339 (15)	0.44001 (9)	0.0347 (6)	
N2	0.41870 (14)	0.40760 (17)	0.49094 (11)	0.0440 (7)	
C1	0.13012 (15)	0.19541 (19)	0.28039 (12)	0.0337 (7)	
C2	0.12955 (16)	0.17534 (19)	0.35014 (13)	0.0374 (7)	
C3	0.13788 (15)	0.27229 (18)	0.39614 (12)	0.0332 (7)	
C4	0.14684 (14)	0.38895 (17)	0.37333 (11)	0.0274 (6)	
C5	0.14494 (16)	0.40667 (18)	0.30168 (11)	0.0348 (7)	
C6	0.13673 (16)	0.3107 (2)	0.25501 (12)	0.0378 (7)	
C7	0.16120 (14)	0.49363 (17)	0.42613 (11)	0.0270 (6)	
C8	0.08175 (14)	0.59034 (17)	0.40049 (10)	0.0274 (6)	
C9	−0.02395 (15)	0.55980 (18)	0.39594 (11)	0.0311 (7)	
C10	−0.10332 (17)	0.6565 (2)	0.37309 (14)	0.0470 (8)	
C11	−0.07795 (17)	0.7496 (2)	0.32396 (14)	0.0465 (8)	
C12	0.02871 (15)	0.80147 (18)	0.35920 (12)	0.0360 (7)	
C13	0.10411 (15)	0.70407 (18)	0.38660 (11)	0.0286 (6)	
C14	0.28249 (15)	0.66677 (18)	0.42763 (10)	0.0285 (6)	
C15	0.26669 (14)	0.54985 (17)	0.44117 (11)	0.0271 (6)	
C16	0.35154 (15)	0.47326 (19)	0.46903 (11)	0.0304 (7)	
H1A	0.42960	0.68370	0.45910	0.0420*	
H1B	0.37370	0.80030	0.42910	0.0420*	
H2	0.12350	0.09570	0.36650	0.0450*	
H3	0.13750	0.25880	0.44450	0.0400*	
H5	0.14940	0.48630	0.28470	0.0420*	
H6	0.13560	0.32360	0.20630	0.0450*	
H7	0.15570	0.46120	0.47300	0.0320*	
H10A	−0.11020	0.69730	0.41680	0.0560*	
H10B	−0.16990	0.61890	0.34740	0.0560*	
H11A	−0.12890	0.81560	0.31430	0.0560*	
H11B	−0.08110	0.71180	0.27700	0.0560*	
H12A	0.04920	0.85040	0.32320	0.0430*	
H12B	0.02750	0.85500	0.39980	0.0430*	

### Atomic displacement parameters (Å^2^)

**Table:**

	*U*^11^	*U*^22^	*U*^33^	*U*^12^	*U*^13^	*U*^23^
Cl1	0.0542 (4)	0.0358 (4)	0.0625 (4)	0.0042 (3)	0.0171 (3)	−0.0181 (3)
O1	0.0357 (8)	0.0312 (9)	0.0545 (10)	−0.0086 (7)	0.0182 (7)	0.0011 (7)
O2	0.0301 (8)	0.0212 (7)	0.0432 (8)	−0.0032 (6)	0.0090 (6)	0.0040 (6)
N1	0.0288 (9)	0.0249 (9)	0.0471 (11)	−0.0069 (7)	0.0070 (8)	0.0050 (8)
N2	0.0314 (10)	0.0353 (11)	0.0633 (13)	−0.0011 (8)	0.0116 (9)	0.0139 (10)
C1	0.0238 (10)	0.0288 (12)	0.0460 (13)	0.0027 (9)	0.0071 (9)	−0.0091 (10)
C2	0.0363 (12)	0.0224 (11)	0.0545 (14)	−0.0011 (9)	0.0155 (10)	0.0015 (10)
C3	0.0345 (12)	0.0275 (11)	0.0383 (12)	−0.0036 (9)	0.0120 (9)	0.0030 (9)
C4	0.0224 (10)	0.0224 (10)	0.0366 (11)	−0.0036 (8)	0.0078 (8)	0.0000 (8)
C5	0.0405 (12)	0.0240 (11)	0.0390 (12)	−0.0003 (9)	0.0111 (10)	0.0028 (9)
C6	0.0410 (12)	0.0350 (13)	0.0359 (12)	0.0044 (10)	0.0095 (10)	−0.0001 (10)
C7	0.0279 (10)	0.0222 (10)	0.0309 (11)	−0.0038 (8)	0.0091 (8)	0.0027 (8)
C8	0.0268 (10)	0.0234 (11)	0.0319 (11)	−0.0009 (8)	0.0089 (8)	−0.0005 (8)
C9	0.0303 (11)	0.0285 (12)	0.0344 (11)	−0.0032 (9)	0.0099 (9)	−0.0045 (9)
C10	0.0290 (12)	0.0374 (13)	0.0759 (18)	0.0014 (10)	0.0182 (11)	0.0005 (12)
C11	0.0323 (13)	0.0334 (13)	0.0691 (17)	0.0046 (10)	0.0085 (12)	0.0082 (12)
C12	0.0365 (12)	0.0261 (11)	0.0441 (13)	0.0016 (9)	0.0104 (10)	0.0042 (10)
C13	0.0282 (11)	0.0261 (11)	0.0316 (11)	−0.0014 (9)	0.0095 (8)	−0.0025 (9)
C14	0.0278 (11)	0.0277 (11)	0.0287 (11)	−0.0018 (8)	0.0066 (8)	−0.0004 (8)
C15	0.0256 (10)	0.0230 (10)	0.0311 (11)	−0.0035 (8)	0.0064 (8)	0.0008 (8)
C16	0.0273 (11)	0.0262 (11)	0.0356 (12)	−0.0077 (9)	0.0067 (9)	0.0032 (9)

### Geometric parameters (Å, º)

**Table:**

Cl1—C1	1.751 (2)	C8—C13	1.343 (3)
O1—C9	1.227 (3)	C9—C10	1.496 (3)
O2—C13	1.376 (3)	C10—C11	1.515 (3)
O2—C14	1.377 (3)	C11—C12	1.528 (3)
N1—C14	1.338 (3)	C12—C13	1.478 (3)
N2—C16	1.150 (3)	C14—C15	1.352 (3)
N1—H1B	0.8800	C15—C16	1.410 (3)
N1—H1A	0.8800	C2—H2	0.9500
C1—C6	1.381 (3)	C3—H3	0.9500
C1—C2	1.372 (3)	C5—H5	0.9500
C2—C3	1.378 (3)	C6—H6	0.9500
C3—C4	1.383 (3)	C7—H7	1.0000
C4—C7	1.518 (3)	C10—H10A	0.9900
C4—C5	1.394 (3)	C10—H10B	0.9900
C5—C6	1.378 (3)	C11—H11A	0.9900
C7—C8	1.502 (3)	C11—H11B	0.9900
C7—C15	1.523 (3)	C12—H12A	0.9900
C8—C9	1.469 (3)	C12—H12B	0.9900
			
C13—O2—C14	118.95 (15)	O2—C14—C15	121.62 (19)
H1A—N1—H1B	120.00	C7—C15—C14	123.71 (18)
C14—N1—H1A	120.00	C7—C15—C16	117.10 (17)
C14—N1—H1B	120.00	C14—C15—C16	119.17 (19)
Cl1—C1—C2	118.92 (16)	N2—C16—C15	177.8 (2)
C2—C1—C6	121.4 (2)	C1—C2—H2	120.00
Cl1—C1—C6	119.64 (17)	C3—C2—H2	120.00
C1—C2—C3	119.1 (2)	C2—C3—H3	119.00
C2—C3—C4	121.3 (2)	C4—C3—H3	119.00
C3—C4—C7	120.28 (18)	C4—C5—H5	119.00
C3—C4—C5	118.24 (18)	C6—C5—H5	119.00
C5—C4—C7	121.46 (17)	C1—C6—H6	121.00
C4—C5—C6	121.16 (19)	C5—C6—H6	121.00
C1—C6—C5	118.8 (2)	C4—C7—H7	108.00
C8—C7—C15	109.00 (16)	C8—C7—H7	108.00
C4—C7—C8	113.00 (17)	C15—C7—H7	108.00
C4—C7—C15	111.14 (16)	C9—C10—H10A	109.00
C7—C8—C9	117.51 (17)	C9—C10—H10B	109.00
C7—C8—C13	123.17 (19)	C11—C10—H10A	109.00
C9—C8—C13	119.15 (18)	C11—C10—H10B	109.00
O1—C9—C8	120.54 (19)	H10A—C10—H10B	108.00
C8—C9—C10	118.15 (18)	C10—C11—H11A	110.00
O1—C9—C10	121.2 (2)	C10—C11—H11B	110.00
C9—C10—C11	112.7 (2)	C12—C11—H11A	109.00
C10—C11—C12	110.5 (2)	C12—C11—H11B	110.00
C11—C12—C13	110.97 (17)	H11A—C11—H11B	108.00
O2—C13—C8	123.17 (18)	C11—C12—H12A	109.00
O2—C13—C12	111.50 (17)	C11—C12—H12B	109.00
C8—C13—C12	125.3 (2)	C13—C12—H12A	109.00
N1—C14—C15	127.46 (19)	C13—C12—H12B	109.00
O2—C14—N1	110.92 (17)	H12A—C12—H12B	108.00
			
C13—O2—C14—N1	−175.33 (16)	C4—C7—C8—C13	−117.7 (2)
C13—O2—C14—C15	4.4 (3)	C15—C7—C8—C9	−168.89 (17)
C14—O2—C13—C8	−2.4 (3)	C15—C7—C8—C13	6.4 (3)
C14—O2—C13—C12	176.27 (17)	C8—C7—C15—C16	177.19 (17)
Cl1—C1—C2—C3	177.03 (18)	C13—C8—C9—C10	2.3 (3)
Cl1—C1—C6—C5	−177.07 (18)	C7—C8—C13—C12	178.03 (19)
C2—C1—C6—C5	1.3 (3)	C9—C8—C13—O2	171.79 (18)
C6—C1—C2—C3	−1.4 (3)	C7—C8—C13—O2	−3.5 (3)
C1—C2—C3—C4	−0.1 (3)	C7—C8—C9—O1	1.2 (3)
C2—C3—C4—C7	−177.0 (2)	C7—C8—C9—C10	177.81 (18)
C2—C3—C4—C5	1.4 (3)	C13—C8—C9—O1	−174.36 (19)
C3—C4—C7—C15	112.8 (2)	C9—C8—C13—C12	−6.7 (3)
C3—C4—C7—C8	−124.3 (2)	O1—C9—C10—C11	−154.9 (2)
C3—C4—C5—C6	−1.5 (3)	C8—C9—C10—C11	28.4 (3)
C5—C4—C7—C8	57.4 (3)	C9—C10—C11—C12	−53.7 (3)
C5—C4—C7—C15	−65.5 (2)	C10—C11—C12—C13	48.8 (2)
C7—C4—C5—C6	176.9 (2)	C11—C12—C13—O2	161.65 (18)
C4—C5—C6—C1	0.1 (3)	C11—C12—C13—C8	−19.7 (3)
C4—C7—C15—C14	120.7 (2)	O2—C14—C15—C7	−0.6 (3)
C4—C7—C15—C16	−57.6 (2)	O2—C14—C15—C16	177.71 (18)
C8—C7—C15—C14	−4.5 (3)	N1—C14—C15—C7	179.11 (19)
C4—C7—C8—C9	67.0 (2)	N1—C14—C15—C16	−2.6 (3)

### Hydrogen-bond geometry (Å, º)

**Table:**

*D*—H···*A*	*D*—H	H···*A*	*D*···*A*	*D*—H···*A*
N1—H1*A*···N2^i^	0.88	2.25	3.132 (3)	177
N1—H1*B*···O1^ii^	0.88	2.15	2.955 (2)	151
C3—H3···N2^iii^	0.95	2.48	3.226 (3)	135

Symmetry codes: (i) −*x*+1, −*y*+1, −*z*+1; (ii) *x*+1/2, *y*+1/2, *z*; (iii) −*x*+1/2, −*y*+1/2, −*z*+1.
